# A note on the seismicity of Sumatra, western Sunda Arc, Indonesia, in relation to the potential for back-arc thrusting

**DOI:** 10.1038/s41598-024-64076-7

**Published:** 2024-06-07

**Authors:** S. Widiyantoro, P. Supendi, N. Rawlinson, M. R. Daryono, S. Rosalia

**Affiliations:** 1 Global Geophysics Research Group, Faculty of Mining and Petroleum Engineering, Institut Teknologi Bandung, Bandung, 40132 Indonesia; 2 Faculty of Engineering, Maranatha Christian University, Bandung, 40164 Indonesia; 3 Department of Earth Sciences – Bullard Labs, University of Cambridge, Cambridge, CB3 0EZ UK; 4 Agency for Meteorology, Climatology and Geophysics, Jakarta, 10720 Indonesia; 5 Research Group for Earthquake Research, Research Centre for Geological Disaster, National Research and Innovation Agency (BRIN), Bandung, 40135 Indonesia

**Keywords:** Natural hazards, Solid Earth sciences

## Abstract

The existence of back-arc thrust faults along the eastern part of the Sunda Arc, ranging westwards from Flores to the western tip of Java, has been recognised for decades. In contrast, it is still unknown whether such back-arc thrust faults exist in Sumatra, which is located in the western part of the Sunda Arc. To investigate the possible existence of back-arc thrusts in Sumatra, we examine regional earthquake data reported by the Agency for Meteorology, Climatology and Geophysics of Indonesia, as well as global earthquake data reported by the International Seismological Centre and the United States Geological Survey. It appears that back-arc thrusts in the study area are not extensively developed, unlike in the eastern Sunda Arc, which may be caused by oblique subduction beneath the Sumatran forearc. The stress associated with the trench-parallel component of subduction is largely accommodated by the ~ 1650-km-long dextral strike-slip fault zone of the Great Sumatran Fault. The seismicity data from various sources do, however, show that there is a dipping seismogenic zone in several parts of the back-arc region of Sumatra, in the opposite direction to the NNE subduction of the Indo-Australian plate. This new observation may be related to the presence of spatially intermittent back-arc thrust faults in the study area, which may need to be taken into account when improving Indonesia's national earthquake hazard maps.

## Introduction

**Figure 1:**
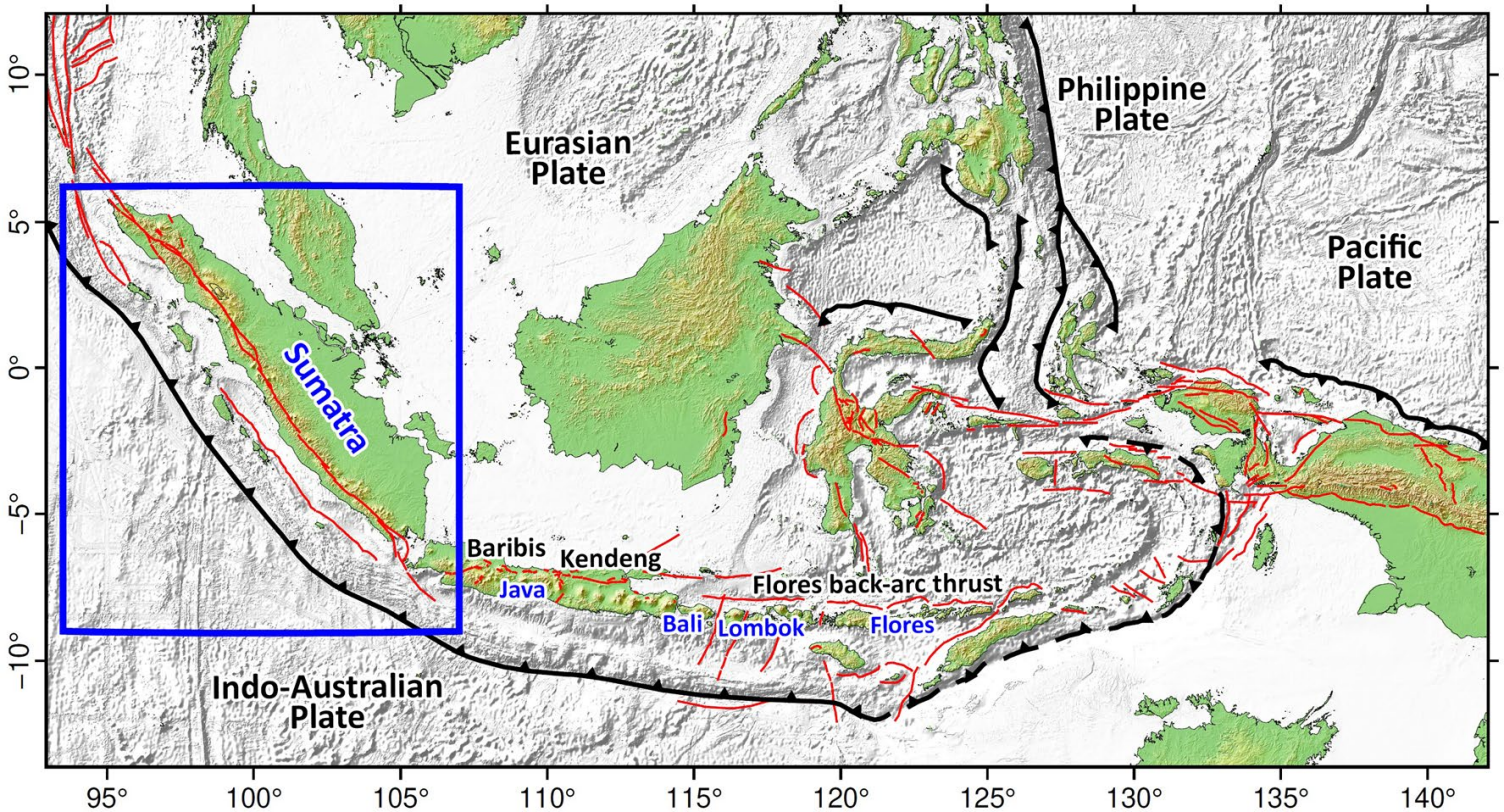
Tectonic setting of the Indonesian region. Solid and dashed black sawtooth lines depict convergent zones/trenches and collision zones, respectively, whereas red lines represent active faults^8^. Note that back-arc thrust faults have been identified from Flores through to Java (Kendeng and Baribis Faults). The ~ 1650-km long, trench-parallel fault on Sumatra is known as the Great Sumatran Fault Zone^9^. The blue rectangle represents our study area. Figure made with Generic Mapping Tools (GMT v.6: https://www.generic-mapping-tools.org).

Back-arc thrusts are thrust faults that lie outboard of volcanic arcs and dip in the opposite direction to the subducting slab. In the Indonesian region, the most well-known back-arc thrust faults are located north of Flores and other islands further west along the eastern part of the Sunda Arc (Fig. 1). Although the back-arc thrusts lie relatively far behind Flores to Bali, further west they are located on land in Java. It is possible that back-arc thrusts in Sumatra are also located on land, since they may represent an extension of the back-arc thrust system that traverses Java.

The Flores back-arc thrust produced a major earthquake of magnitude 7.9 that generated a devastating tsunami on December 12, 1992^1–5^. More recently, another major event related to Flores back-arc thrust activity occurred further west, and was followed by a series of earthquakes in northern Lombok in 2018^6,7^. From seismicity studies, Widiyantoro and Fauzi^10^ show that the Flores back-arc thrust system also extends to the north of Bali, and even further west through eastern Java to western Java^11,12^. In eastern Java, the back-arc thrust is known as the Kendeng thrust fault system^8,13, 14^, whereas in western Java it is known as the Baribis Fault^12,15, 16^. The Baribis Fault system has recently been referred to as the northwest Java back‐arc thrust by Aribowo et al.^11^.

A question that is frequently asked is whether a back-arc fault system exists along Sumatra that might be similar in scale to what is observed in the eastern section of the Sunda Arc. The change from typical trench-perpendicular subduction along the eastern Sunda Arc to oblique subduction along Sumatra in the western part of the Sunda Arc^17^ may explain the apparent absence of back-arc thrusts in Sumatra. It is estimated that the trench-normal component of convergence is ~ 45 mm/yr, and the trench parallel component is ~ 29 mm/yr^18^. The latter component is largely accommodated by slip on the Great Sumatran Fault, which comprises a complex network of on-shore and offshore strike-slip faults, some of which appear to be locked^19^. Furthermore, there is evidence of convergent strain being accommodated by backstop faults in some parts of the forearc^20^, which adds to the difficultly of understanding how evenly stress is distributed in the back-arc region.

This study aims to search for evidence of active thrust faults in the back-arc region of Sumatra. To do so, we identify regions where seismic catalogs record thrust earthquakes, which may coincide with evidence of surface shortening, such as folding that is related to ongoing back-arc thrusting. Although the available data are currently limited, we bring together constraints from various modern earthquake catalogues, our own analysis of earthquake focal mechanisms, and evidence from surface mapping. The discovery of active back-arc thrusts would significantly change our understanding earthquake hazard and risk in northeast Sumatra, which has implications for the development of quantitative earthquake hazard maps and the distribution of stations in Indonesia’s national seismic network, which are currently focused in the forearc region above the megathrust.

To conduct the investigation, we used data sourced from the Indonesian Tsunami Early Warning System (InaTEWS) catalog reported by BMKG, together with data from the International Seismological Centre Engdahl, Hilst, Buland catalogue (ISC-EHB), the Global Earthquake Model (ISC-GEM) catalogue and the USGS catalog. The differences between these catalogues, including their relative advantages and disadvantages, are described in the MATERIALS AND METHODS section. Interestingly, all these catalogs consistently contain seismicity that can be associated with back-arc thrusting.

To the best of our knowledge, the possible existence of a back-arc thrust system along Sumatra has not been published elsewhere, except for a portion in northern Sumatra reported by Muksin et al.^21^, who found evidence of a secondary fault system based on recent seismicity and geomorphic structure in the region. Therefore, the results of this study may be of particular significance for better understanding the natural hazards faced by Sumatra’s large and growing population of 60 million people.

## Results and discussion

**Figure 2:**
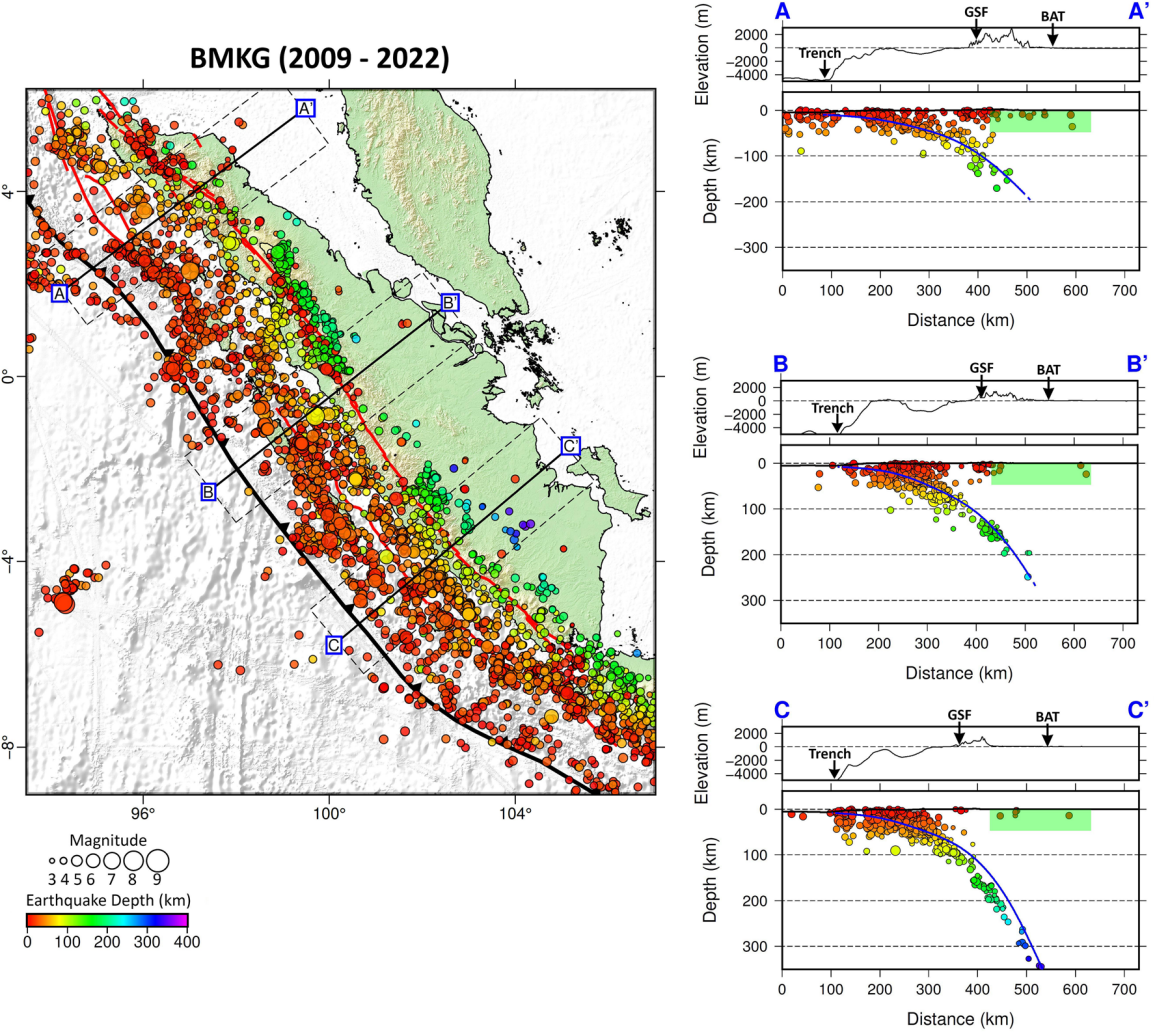
Plots of relocated hypocenters from BMKG (2009 – 2022) with magnitude > 2.0. The locations of cross sections A–C are shown in map view on the left. Vertical cross sections of USGS earthquakes are displayed on the right. Note that the number of events is reduced due to relocation criteria. Coloured dots represent hypocenters projected from a distance of up to 50 km on either side of the cross-section. Blue lines depict the plate interface of the subducted Indo-Australian Plate according to the Slab2.0 model^22^. Regions that have shallow events (as approximately indicated by the green shaded areas on the vertical cross sections) are interpreted as zones that can accommodate back-arc thrusts. Abbreviations: Great Sumatran Fault (GSF) and back-arc thrust (BAT). Figure made with Generic Mapping Tools (GMT v.6: https://www.generic-mapping-tools.org).

**Figure 3:**
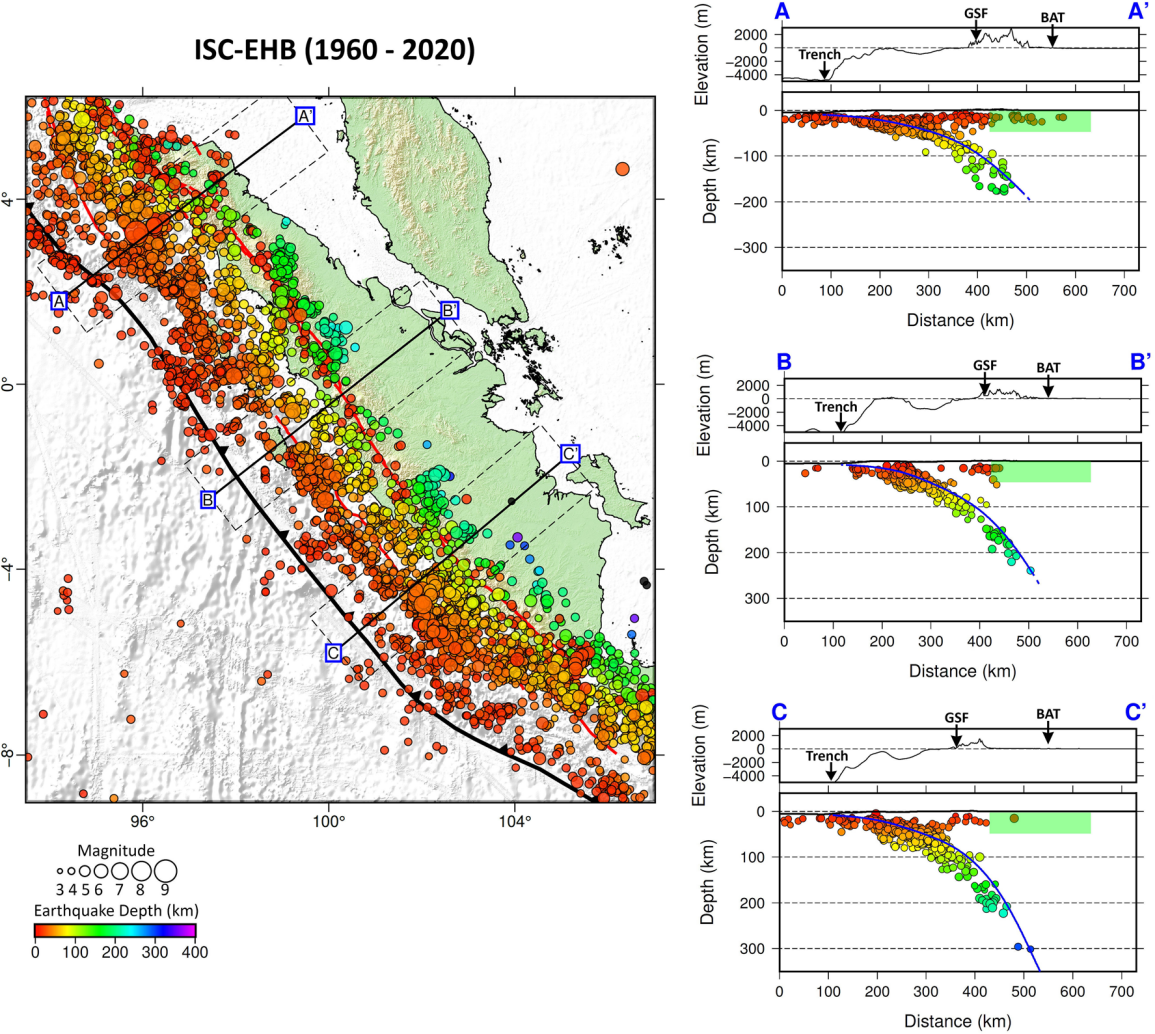
Plots of relocated hypocenters from ISC-EHB (1960 – 2020) with magnitude > 4.0. Note that the number of events is significantly reduced due to hypocenter relocation criteria. As in Fig. 2, coloured dots represent hypocenters projected from a distance of up to 50 km on either side of the cross-section. Blue lines depict the plate interface of the subducted Indo-Australian Plate according to the Slab2.0 model^22^. Regions that have shallow events (as approximately indicated by the green shaded areas on the vertical cross sections) are interpreted as zones that can accommodate back-arc thrusts. Abbreviations: Great Sumatran Fault (GSF) and back-arc thrust (BAT). Figure made with Generic Mapping Tools (GMT v.6: https://www.generic-mapping-tools.org).

The result of our seismicity investigation, based on data taken from the BMKG catalogue (2009–2022), which have earthquakes of magnitudes > 2.0 that were detected by Indonesia’s national network, reveals seismic events concentrated along the Wadati–Benioff zone beneath Sumatra, as shown in Fig. 2 (right panel). Interestingly, there are shallow events, some at depths of less than 50 km, northeast of the subduction zone, that are worthy of further investigation in the context of back-arc thrust faults. Several of these events are located away from the Great Sumatran Fault (GSF), as seen in map view and vertical cross sections A–A′, B–B′, and C–C′ (the green shaded areas) in Fig. 2, and may reflect back-arc thrusting. In Fig. 3, we plot earthquakes from the ISC-EHB global catalog (1960–2020) that are of magnitude > 4.0. While the number of events is limited in comparison to the BMKG catalogue, due to strict hypocenter relocation criteria, we can still clearly observe several shallow events that can be correlated with possible back-arc thrusting. In particular, we seek to identify events in the back-arc region (NE Sumatra) that exhibit thrust mechanisms and have fault planes that strike parallel to the trench and dip to the SW. The back-arc thrust-like feature observed in Figs. 2 and 3 is also consistently depicted by the seismicity plot from global catalogs, namely the USGS catalog and the ISC-GEM catalog shown in Supplementary Figs. 1 and 2.

**Figure 4:**
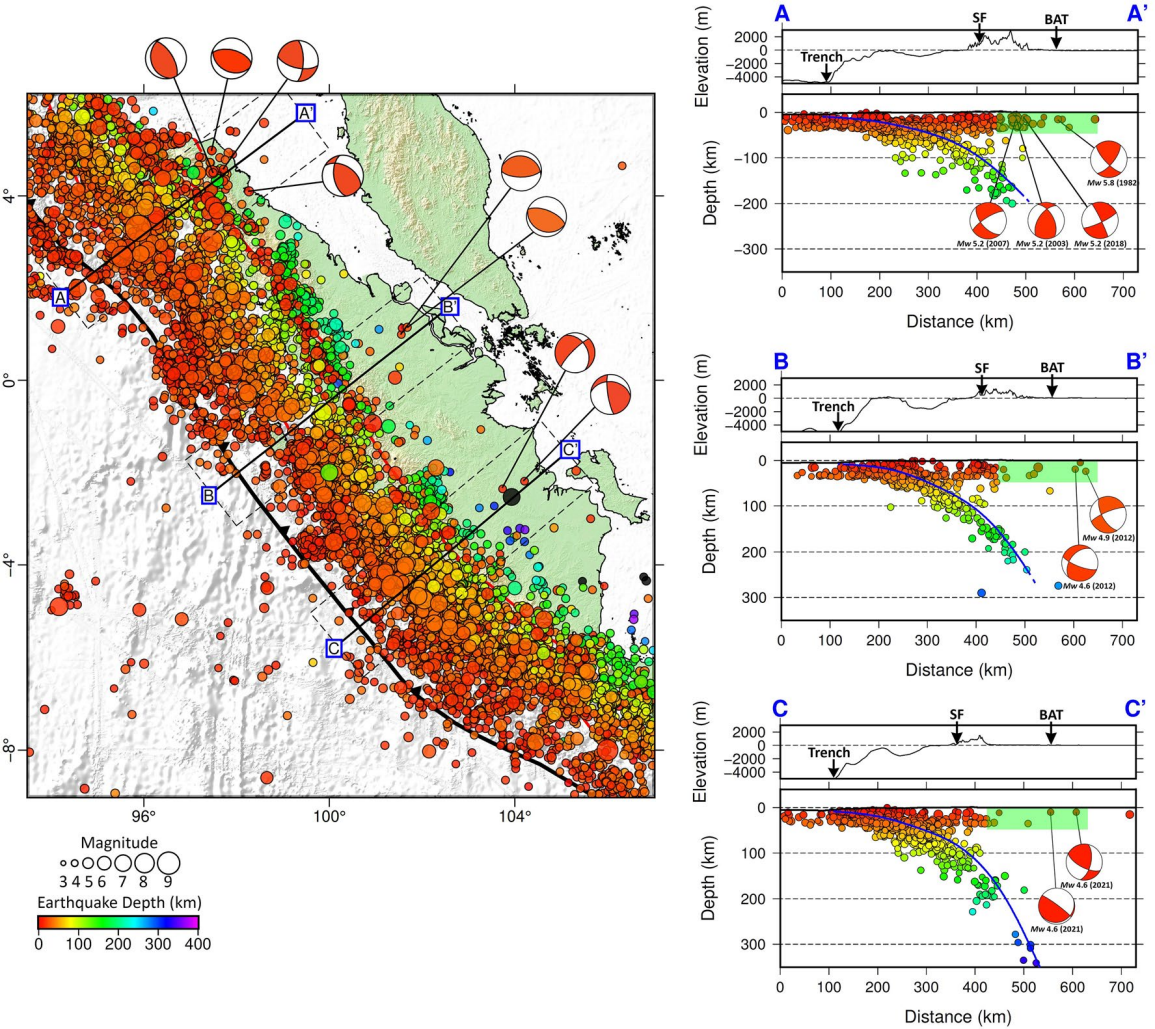
Plots of hypocenters from PUSGEN’s compiled catalogs for events with Mw ≥ 4.5. It should be noted that the compilation of multiple catalogs results in a notable increase in the number of recorded seismic events. Focal mechanisms of selected events derived using BMKG data (Table 1) are shown in horizontal and vertical sections. They show a thrust mechanism in the back-are region, with a strike-slip component. Focal mechanism data taken from the global CMT catalogue (globalcmt.org) for Sumatra in the period from 1976 to 2023 is presented in Supplementary Figs. 3 and 4. As in Figs. 2 and 3, coloured dots represent hypocenters projected from a distance of up to 50 km on either side of the cross-section. Blue lines depict the plate interface of the subducted Indo-Australian Plate according to the Slab2.0 model^22^. Regions that have shallow events (as approximately indicated by the green shaded areas on the vertical cross sections) are interpreted as zones that can accommodate back-arc thrusts. Abbreviations: Great Sumatran Fault (GSF) and back-arc thrust (BAT). Figure made with Generic Mapping Tools (GMT v.6: https://www.generic-mapping-tools.org).

Figure 4 includes earthquake locations from catalogs compiled by the National Center for Earthquake Studies (PUSGEN) of the Ministry of Public Works and Housing (PUPR), Indonesia. In this figure, we plot the focal mechanisms of selected events from the Global Centroid-Moment-Tensor (CMT) catalogue and those derived from BMKG data. They suggest thrusting in the back-arc region, especially in north, central and south Sumatra (see the map view and the vertical cross sections A–A′, B–B′, and C–C′). This allows us to assess the potential for back-arc thrust faults in the study region in more detail, as discussed below.

Silver et al.^23^ explain that several factors can promote or hinder the development of thrust faults. One is crustal weakness arising from the thermal impact of magmatic intrusion, which facilitates greater deformability in the Earth's crust. Another factor is the slope of the surface, although it is not considered the primary driving force. In the context of the eastern Sunda arc, Silver et al.^23^ report that the primary cause of back-arc thrust faulting is collision between the arc and continent. It is envisaged that the collision of the Banda arc and the Australian continent represents the dominant mechanism acting to drive back-arc thrusting north of Flores. McCaffrey and Nabelek^24^ who investigate the geometry of back-arc thrusting along the eastern Sunda Arc using earthquake and gravity data, suggest that the Flores thrust represents the surface expression of a deep-seated thrust zone. Above the main back-arc thrust fault there are a series of imbricate or overlapping thrust faults. These imbricate thrust faults are shallower in depth than the main Flores back-arc thrust (see e.g.^25,26^). They show that the Flores back-arc thrust faults dipping in the opposite direction to the main subduction of the Indo-Australian plate down to a depth of approximately 40–50 km.

**Figure 5:**
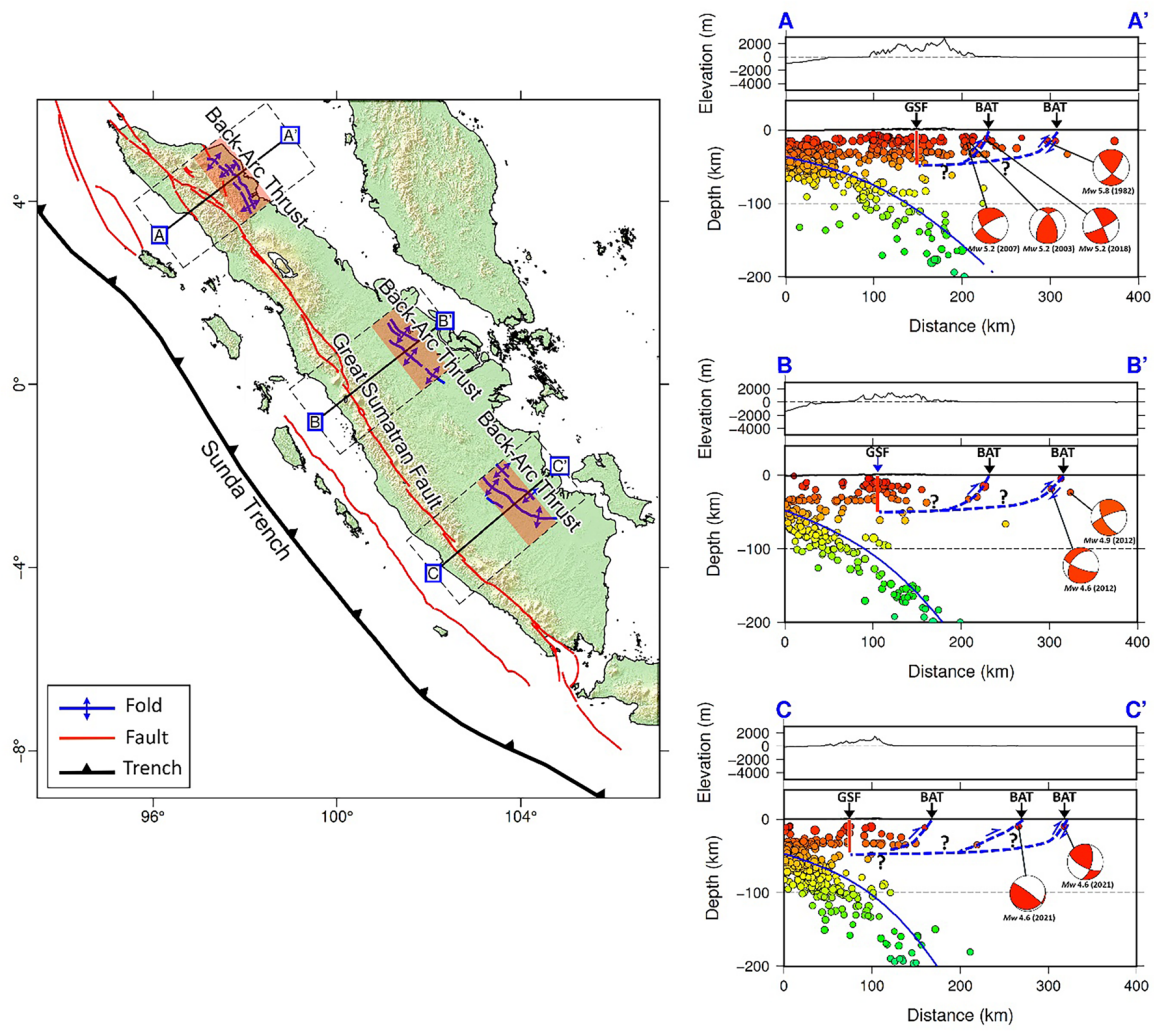
Summary map of Sumatra that shows the location of possible back-arc thrusts according to our interpretation. Blue lines in map view depict the location of folds, which coincide with possible back-arc thrusts in north, central and south Sumatra; the red shaded areas are the approximate locations of back-arc regions plotted on a FABDEM topographic map^27^) (left). Panels on the right show close-ups of vertical cross sections in Fig. 4 with the interpretation of possible back-arc thrusts indicated by blue dashed-lines (right). Question marks indicate that the presence of the shallow dipping decollement is not yet supported by any data observations. Abbreviations: Great Sumatran Fault (GSF) and back-arc thrust (BAT). Figure made with Generic Mapping Tools (GMT v.6: https://www.generic-mapping-tools.org).

In the case of Sumatra, it appears that it lacks a coherent or extensive back-arc thrust fault system, which may be associated with the oblique subduction of Sumatra^17^. However, upon closer examination, several focal mechanisms of events that occur in the Sumatra back-arc region, displayed in Fig. 4, as well as global CMT solutions shown in Supplementary Figs. 3 and 4, depict SSW-dipping zones when strikes of nodal planes < 180° are chosen (see Table 1). Our analysis is summarized by the map and close ups of vertical cross sections shown in Fig. 5, which is largely based on the focal mechanisms of selected events, along with the geological map of Sumatra from Gafoer et al.^28,29^ and morphology based on the topographic map from FABDEM V1-2 (Forests and Buildings removed Copernicus DEM) that removes building and tree height biases from the Copernicus GLO 30 Digital Elevation Model (DEM) provided by Neal & Hawker^27^ displayed in Supplementary Figs. 6–8 (with locations shown in Supplementary Fig. S5). Assuming that the nodal plane corresponding to back-arc thrusts dips in the opposite direction to the subducting Indo-Australian plate, it has an average strike of 146° and average dip angle that varies from 45°, 46° and 56° in north, central, and south Sumatra, respectively (Table 1). We found that the location of the back-arc thrusts approximately coincides with the folds of northern, central, and southern Sumatra (Fig. 5). These characteristics are similar to active back-arc thrust faults in Java (see^30,31^. This finding deserves further investigation, such as detailed geological mapping as conducted by Aribowo et al.^11^ in northwest Java, and geophysical investigations, for instance using borehole seismometers (see^12^), and carrying out seismic tomography using local earthquake data. In the latter case, we need to add more stations, especially within the back-arc region to resolve structures related to back-arc thrusting; currently, most of the stations are placed along the arc, with relatively few in the back-arc region (Supplementary Fig. 9, left). These additional stations would also improve seismic tomography in the back-arc region (Supplementry Fig. 9, right) which may help in the identification and characterization of active faults.

**Table 1:** Focal mechanisms of selected events based on Global CMT (A–A′) and BMKG data (B–B′ and C–C′).

Cross-section	Date	Time (UTC)	Lat (deg)	Long (deg)	Depth (km)	Mw	Strike (deg)	Dip (deg)	Rake (deg)
A–A'	21/07/2007	12:53:03	5.14	97.72	12	5.2	116	35	100
	13/09/2003	20:42:23	4.74	97.57	15	5.2	103	62	25
	26/09/2018	17:51:43	4.58	97.72	12	5.2	166	27	106
	24/02/1982	04:22:47	4.1	98.27	11	5.8	171	54	126
	Average						139	45	89
B–B′	29/03/2012	12:13:43	1.13	101.57	5	4.6	89	65	84
	31/03/2012	03:58:20	1.15	101.68	24	4.9	111	26	89
	Average						100	46	87
C–C′	4/10/2021	06:20:12	− 2.35	103.73	10	4.6	225	79	-54
	29/11/2021	09:07:44	− 2.18	104.21	10	4.6	171	79	124
	Average						198	79	35
Average for all cross-sections							146	56	70

In summary, it appears that back-arc thrust faults are neither extensively developed nor widespread in Sumatra, most likely due to oblique subduction that generates extensive strike slip fault systems and backstop thrusts in the forearc region rather than a pronounced back-arc thrust fault system. On the other hand, the presence of several seismogenic zones with an opposite dip direction to the subducting slab and similarly dipping thrust focal mechanisms beneath Sumatra suggests that back-arc faults are not entirely absent. However, they are less well developed compared to the eastern part of the Sunda Arc, and are intermittent rather than continuous along strike of the trench. One of the main implications of our results is that further work needs to be done to investigate the presence of back-arc thrust faults in Sumatra, such as microseismic monitoring, crustal seismic tomographic imaging, and detailed geological mapping that includes trenching. The latter technique involves the excavation of trenches to collect geological data and samples in order to find evidence of recently active faults. GPS monitoring can also be used to detect deformation in the back-arc region that may be related to back-arc faulting. If the presence of even localised back-arc thrusting is confirmed, then it may have a considerable impact on the next generation of probabilistic seismic hazard models of Sumatra.

## Materials and methods

### Data sources

The earthquake data used in this study were taken from the regional BMKG earthquake catalog, and the global USGS and ISC archives. The ISC data set we used comes from two catalogs, i.e., ISC-GEM and ISC-EHB. These four earthquake catalogs are useful for carefully investigating back-arc thrust faults in Sumatra.

### BMKG earthquake catalog

The BMKG earthquake data we use are taken from the Indonesian Tsunami Early Warning System (InaTEWS) catalog reported by BMKG. InaTEWS has been in operation since November 2008 and is continuously maintained (http://inatews2.bmkg.go.id/). Events are periodically relocated using a teleseismic double-difference (teletomoDD) algorithm that is an extension of the DD tomography method ^32^ to teleseismic distances^30^. Following Pesicek et al.^33^, Nugraha et al.^34^ and Supendi et al.^35^, the initial seismic velocity model is fixed and the relocation capabilities are used. Travel times are calculated using a 3-D regional seismic velocity model of the Indonesian region with a grid size of 1° × 1°^36^ and the global 1-D model ak135^37^ for regions outside of Indonesia. For ray tracing, a pseudo-bending method^38^ adapted for use in a spherical coordinate system^39^ is employed^35^.

### USGS earthquake catalog

The USGS earthquake catalog contains locations, magnitudes, phase arrival times, and amplitude measurements for earthquakes detected by the National Earthquake Information Center (NEIC), as well as those detected by contributing US regional and foreign networks, including smaller events (https://www.usgs.gov). The NEIC global earthquake bulletin is called the Preliminary Determination of Epicenters (PDE). The word "preliminary" is used because the International Seismological Center (ISC) bulletin is usually regarded as the final global archive of parametric earthquake data, as described below.

### ISC earthquake catalog

Earthquake catalogues represent the primary output of the International Seismological Centre, UK, which is considered the definitive record of seismicity worldwide (http://www.isc.ac.uk/iscbulletin/). This catalog contains data from 1900 to the present day. The reviewed catalog, which is manually checked by ISC analysts, is typically 2 years behind real time. The creation of the catalog depends on data contributed by seismological agencies from all over the world. These data, which include hypocenters, are automatically grouped into events, which form the basis of the ISC catalog. An automatic thresholding process decides whether each earthquake should be manually reviewed by an ISC analyst, which will also be relocated by the ISC depending on data availability.

### ISC-GEM earthquake catalog

The ISC-GEM catalog is the result of a distinct effort to adapt, extend and significantly improve existing bulletin data for large earthquakes (magnitude ≥ 5.5, as well as continental events up to magnitude 5.0) to meet the needs of the specific user group who analyses and models seismic hazard and risk (http://www.isc.ac.uk/iscgem/).

In addition, the catalog also has multi-disciplinary applications in a wide range of other areas, for example: studies of global seismicity, tectonics, inner structure of the Earth, nuclear test monitoring research, and rapid determination of hazard, in which all the magnitudes of earthquakes are already in Mw. This global instrumental earthquake catalog was designed to serve as a reference to be used for calibration purposes by those compiling regional seismicity catalogs, such as the BMKG catalog, that contain events of much smaller magnitude. In this way, catalogs prepared by other teams for different regions will contain comparable earthquake locations and magnitude parameters, particularly in border regions.

### ISC-EHB earthquake catalog

The ISC-EHB catalog is a groomed version of the ISC-reviewed data set, and contains 199,578 seismic events from 1964 to 2020, where the period 1964–2008 has been rebuilt by Engdahl et al.^40^ (http://www.isc.ac.uk/isc-ehb/). Teleseismically well-constrained events are selected from the ISC catalog. To minimise errors in location, especially depth, due to assumed 1D Earth structure, earthquake hypocenters are relocated using the EHB algorithm^41^. To do this, seismic events are selected based on strict criteria, in which the EHB algorithm incorporates a specific phase identification algorithm for teleseismic depth phases, namely pP, pwP, sP and PcP, as well as PKiKP, PKPdf, PKPbc and PKPab.

### PUSGEN’s compiled catalogs

In preparation for updating the present Indonesian national hazard maps, PUSGEN has collected data from various catalogs from 1905 to 2022 with the following priorities for the same event: (1) ISC-EHB and EHB catalog (1960–2020), (2) ISC-GEM catalog (1905–2019), (3) relocated BMKG data (April 2009–2022), (4) reviewed ISC data based on the Global Centroid-Moment-Tensor solutions (1977–2021), and (5) USGS catalog (1905–2022). To standardize the magnitude measurements within the catalog, the methodology outlined by Scordilis^42^ was adopted, which involves formulating equations for converting M_S_ and m_b_ to moment magnitude (Mw).

### Focal mechanisms

Since there are no available focal mechanism data for magnitudes less than 5.0 in the Sumatra back-arc region between 1976 and 2023 (cross-section B–B′ and C–C′), we used MTTime to calculate moment tensors, which is a Python-based time-domain moment tensor inversion code from Chiang^43^. This method uses a time-domain generalized least squares inversion technique ^44^ to match synthetic and observed waveforms. The waveform data from BMKG seismic stations in Sumatra were instrument-corrected to ground displacement, rotated to the great-circle path, decimated to one sample per second, and filtered. We band-pass filtered the seismograms in the frequency range 0.04–0.09 Hz prior to inversion. In the step involving the generation of synthetic seismograms, we adopted a one-dimensional (1-D) seismic velocity model derived from CRUST 1.0^45^ in the region of interest. Crust 1.0 is a global model that divides the crust into 8 layers of variable thickness on a 1 × 1 degree grid; these layers include water, ice, sediments (upper, middle, lower) and crust (upper, middle, lower) and are defined by Vp,Vs and density. A 1-D reference model derived from Crust 1.0 in the region of interest makes it more aligned with local structure than a global reference model. Overall, MTTime was chosen because it allows for full waveform inversion to constrain moment tensor solutions, and has been successfully used in other studies, including those that involve subduction zone settings (e.g.^46^).

The resulting focal mechanisms are crucial in indicating the type of slip that occurs during an earthquake i.e. thrust, normal, strike-slip, or a combination thereof. In addition, focal mechanisms also reveal the strike and dip of the fault that slipped, Since our goal is to find evidence of back-arc thrust faults, any earthquake that lies in the back-arc region, has a thrust mechanism and a fault plane that strikes parallel to the subduction zone and dips toward it, is a potential candidate for rupturing a back-arc thrust fault. When such events cluster, the potential for the existence of an active backarc fault system increases.

### Supplementary Information

Supplementary Information.

## Data Availability

Relocated events from the BMKG catalogue that were used in this study can be downloaded from 10.5281/zenodo.10666694. USGS event location data are freely available at https://www.usgs.gov, whereas ISC-GEM and ISC-EHB event location data are freely available at http://www.isc.ac.uk.
