# Supplementary material for: A note on the seismicity of Sumatra, western Sunda Arc, Indonesia, in relation to the potential for back-arc thrusting
Source: Sci Rep. 2024 Jun 7;14:13115. doi: 10.1038/s41598-024-64076-7 (PMC11161522; doi:10.1038/s41598-024-64076-7)
Supplement: Supplementary file 1 — Supplementary Information. [file 41598_2024_64076_MOESM1_ESM.pdf]

Supplementary information to the manuscript

A note on the seismicity of Sumatra, western Sunda Arc, Indonesia, in relation to the potential for back-arc thrusting

S. Widiyantoro, P. Supendi, N. Rawlinson*, M. R. Daryono and S. Rosalia

*Corresponding author, e-mail: nr441@cam.ac.uk

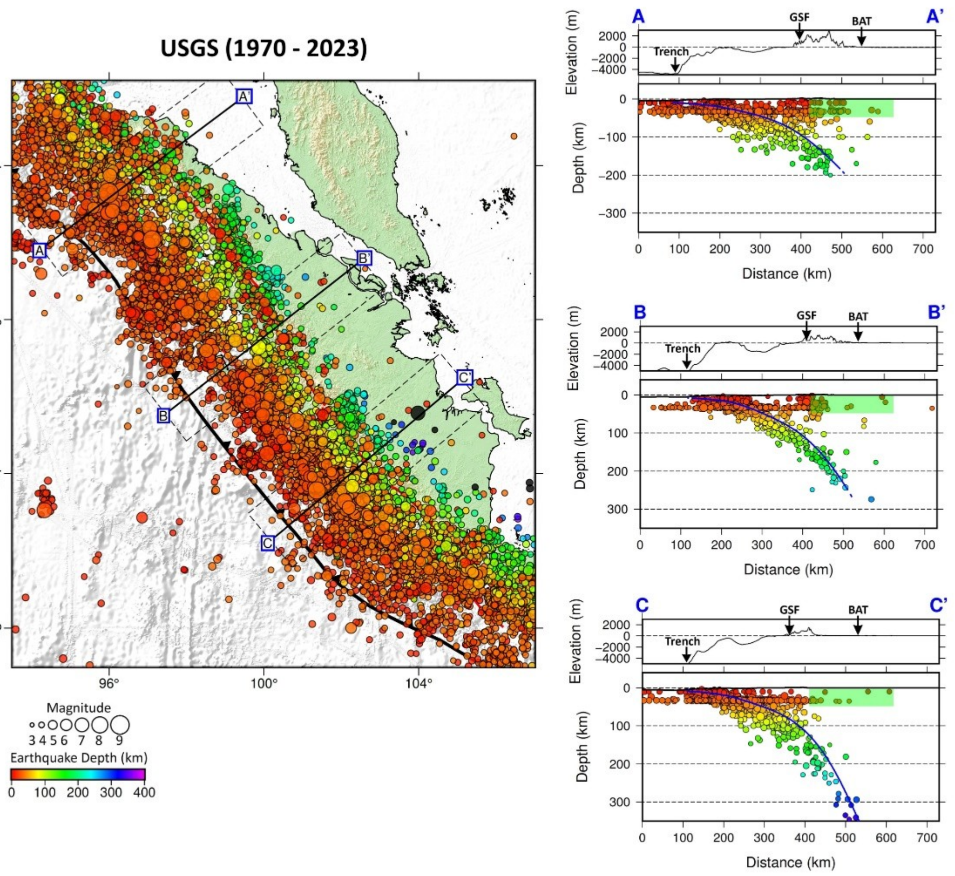

Supplementary Figure 1. *Plots of earthquake data with magnitude > 3.5 reported by the USGS from 1970 to 2023. The locations of cross sections A – C are shown in map view on the left. Vertical cross sections of USGS earthquakes are displayed on the right. Coloured dots represent hypocenters projected from a distance of up to 50 km on either side of the cross-section. Blue lines depict the plate interface of the subducted Indo-Australian Plate according to the Slab2.0 model of Hayes (2018) [22]. Regions that have shallow events (as approximately indicated by the green shaded areas on the vertical cross sections) are interpreted as potentially hosting back-arc thrusts. Abbreviations: Great Sumatran Fault (GSF) and back-arc thrust (BAT).*

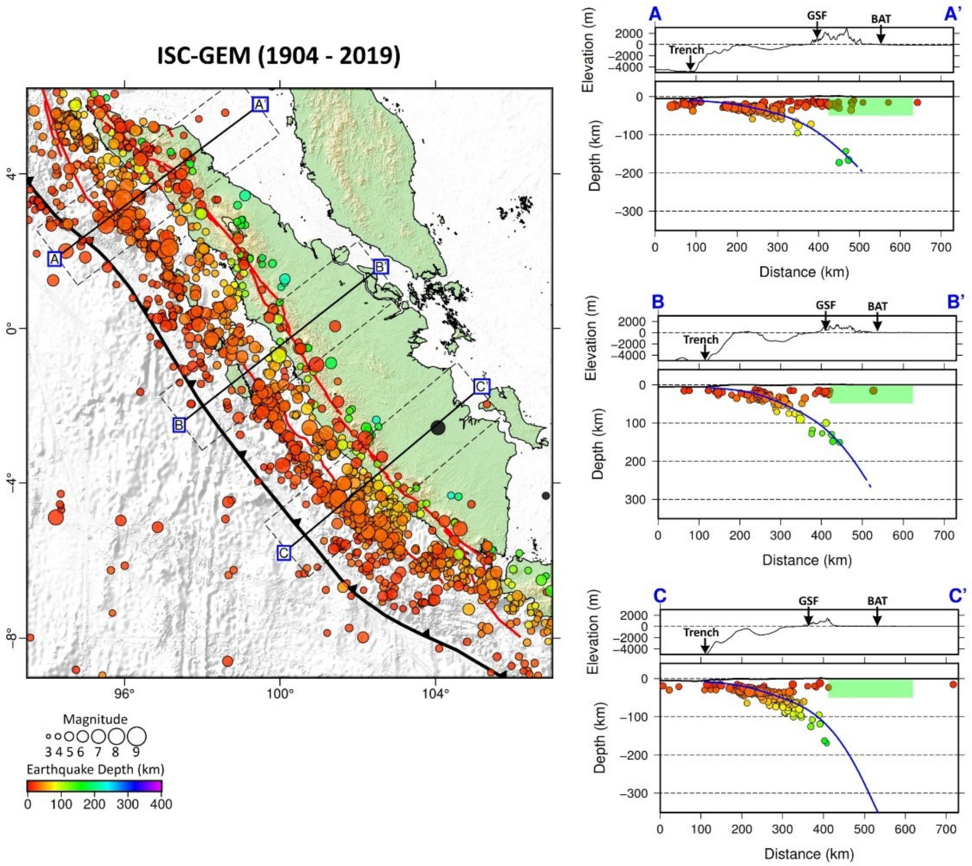

Supplementary Figure 2. *Same as Supplementary Fig. 1 but using earthquake data reported by the ISC-GEM Global Instrumental Earthquake Catalogue (1904 - 2019). Note that the number of events in the ISC-GEM catalog is smaller than in the USGS catalog (see Supplementary Fig. 1), since only events with magnitude > 4.8 are used. As in Supplementary Fig. 1, coloured dots represent hypocenters projected from a distance of up to 50 km on either side of the cross-section. Blue lines depict the plate interface of the subducted Indo-Australian Plate according to the Slab2.0 model (Hayes 2018) [22]. Regions that have shallow events (as approximately indicated by the green shaded areas on the vertical cross sections) are interpreted as potentially hosting back-arc thrusts. Abbreviations: Great Sumatran Fault (GSF) and back-arc thrust (BAT).*

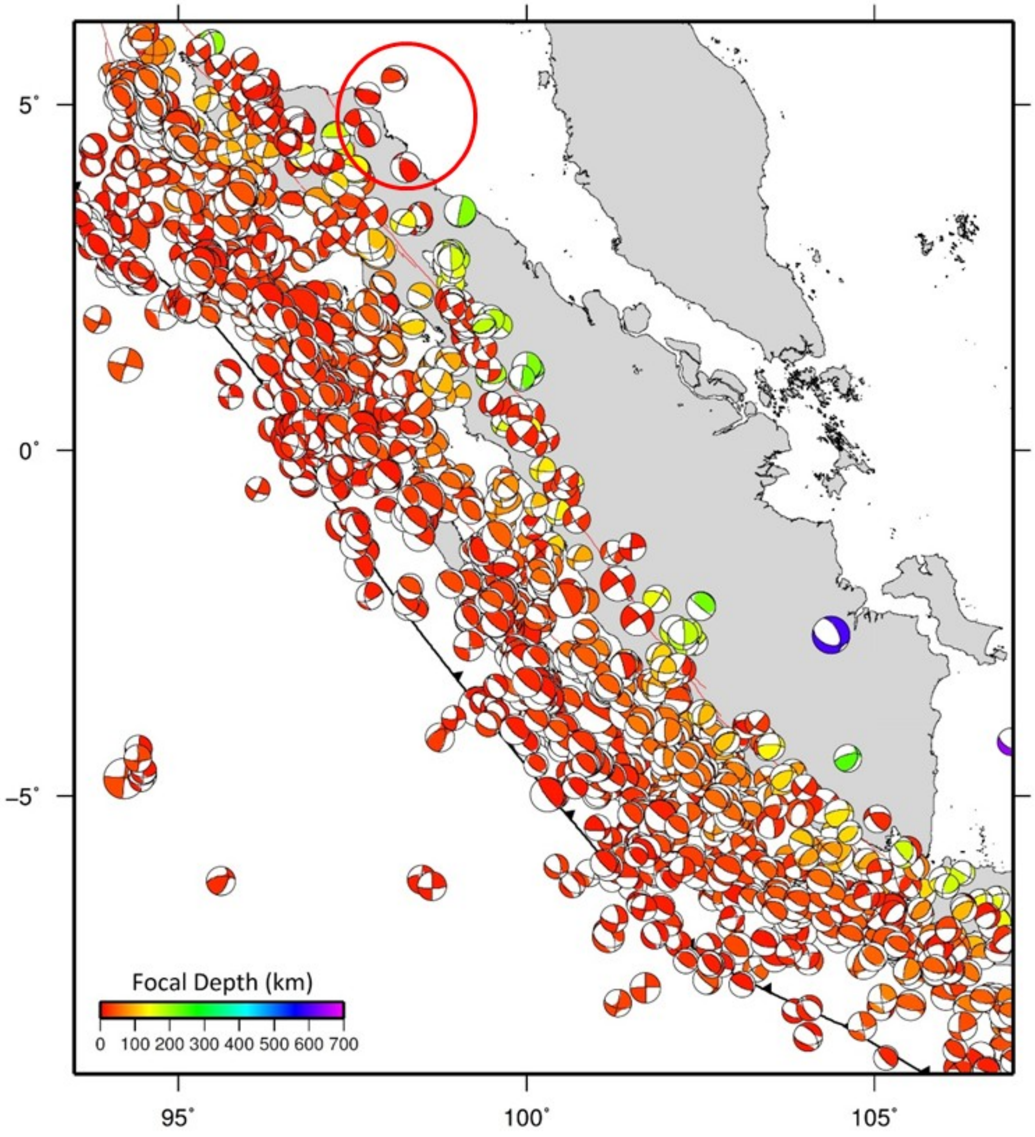

Supplementary Figure 3. *Focal mechanism data taken from the global CMT catalogue (globalcmt.org) for the period from 1976 to 2023 for all depths (Dziewonski et al. 1981 [49] and Ekström et al. 2012 [50]). Note the thrust events in the back-arc region of north Sumatra in the red circle.*

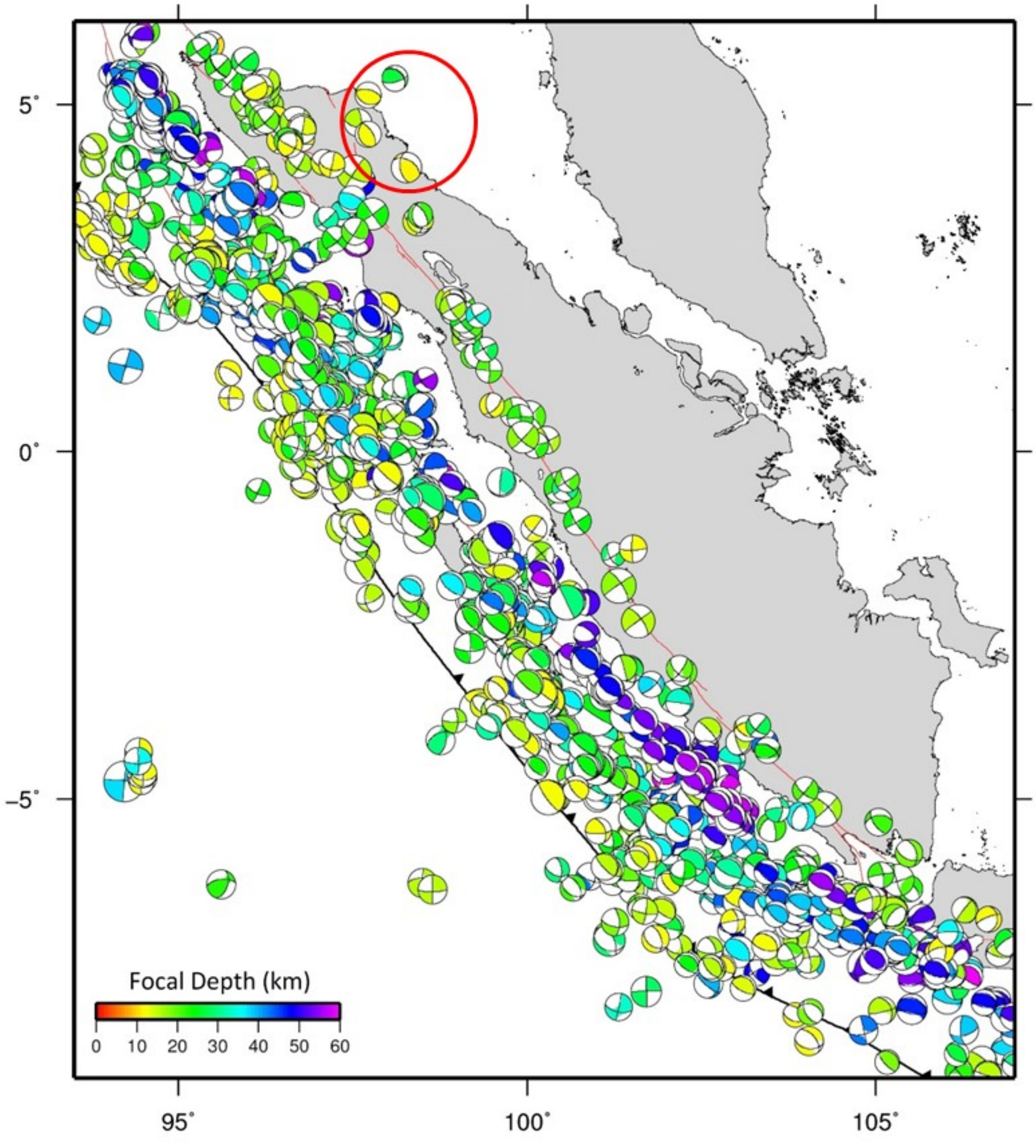

Supplementary Figure 4. *Same as Supplementary Fig. 3, but for depths ≤ 60 km (Dziewonski et al. 1981 [49] and Ekström et al. 2012 [50]). Note the thrust events in the back-arc region of north Sumatra in the red circle.*

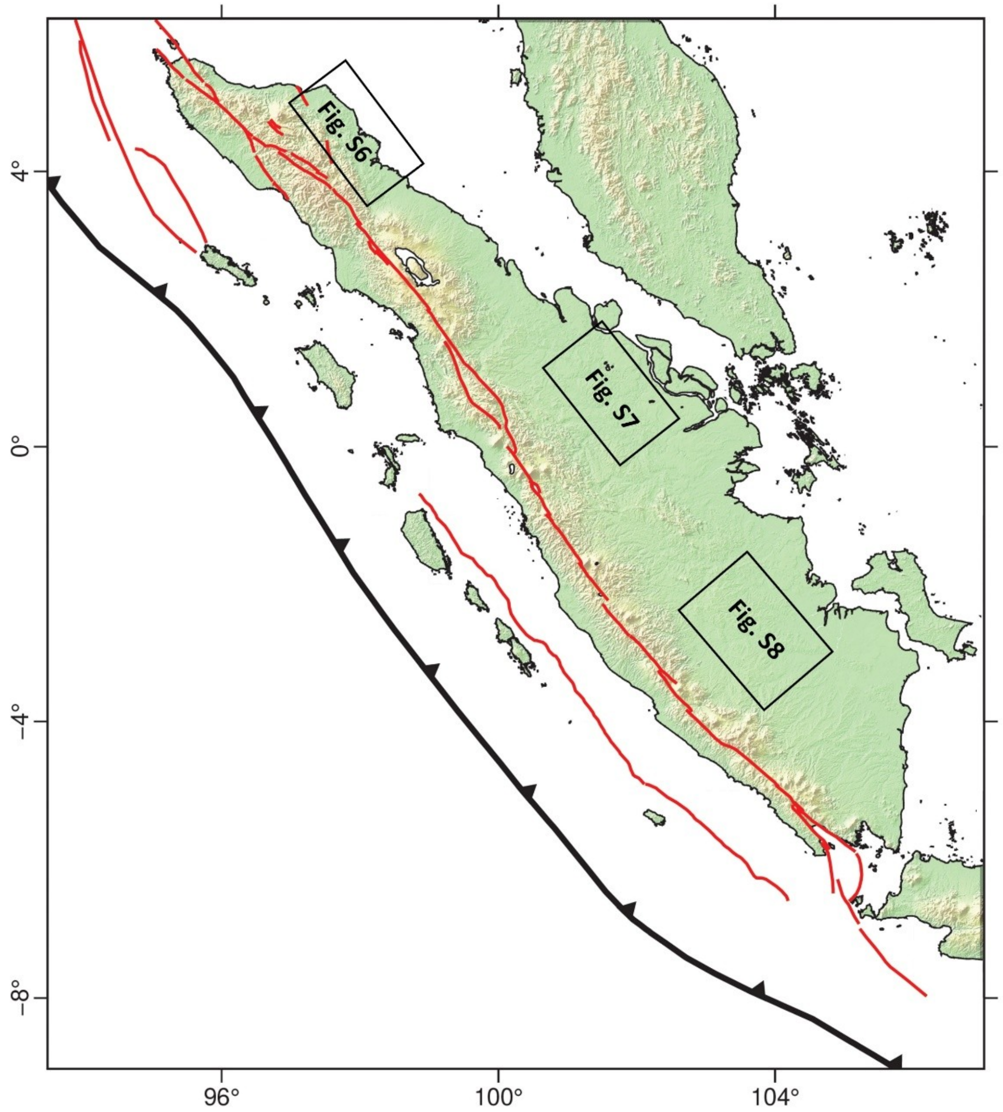

Supplementary Figure 5. *The location of topographic and geological maps shown in Supplementary Figs. 6-8.*

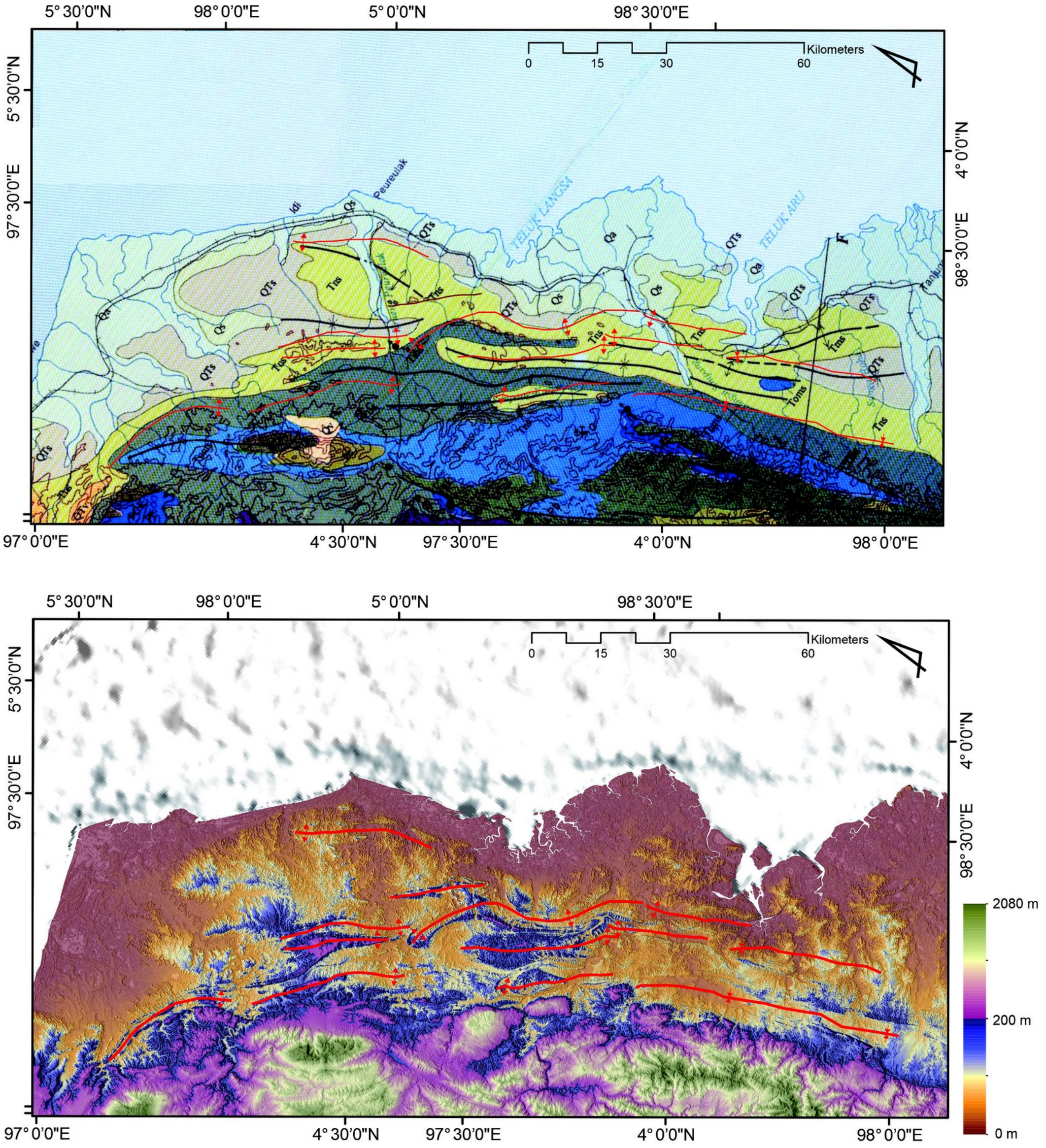

Supplementary Figure 6. *A geological map of northern Sumatra (Gafoer et al. 1996 [29]) (top); and FABDEM topographic map (Neal & Hawker (2023) [27]) (bottom). Red lines depict interpreted folds in the region. See Supplementary Figure 5 for location of map. Qa – Alluvium; Qs - Swamp Deposits; QTs - Plio-Pleistocene Non Marine Sediments; Tns - Mio-Pliocene Transitional Sediments; Tms (dark green) - Miocene Shallow Marine Sediments; Toms (blue) - Oligo-Miocene Transgressive Sediments. Basemaps from Gafoer et al. (1992) [28].*

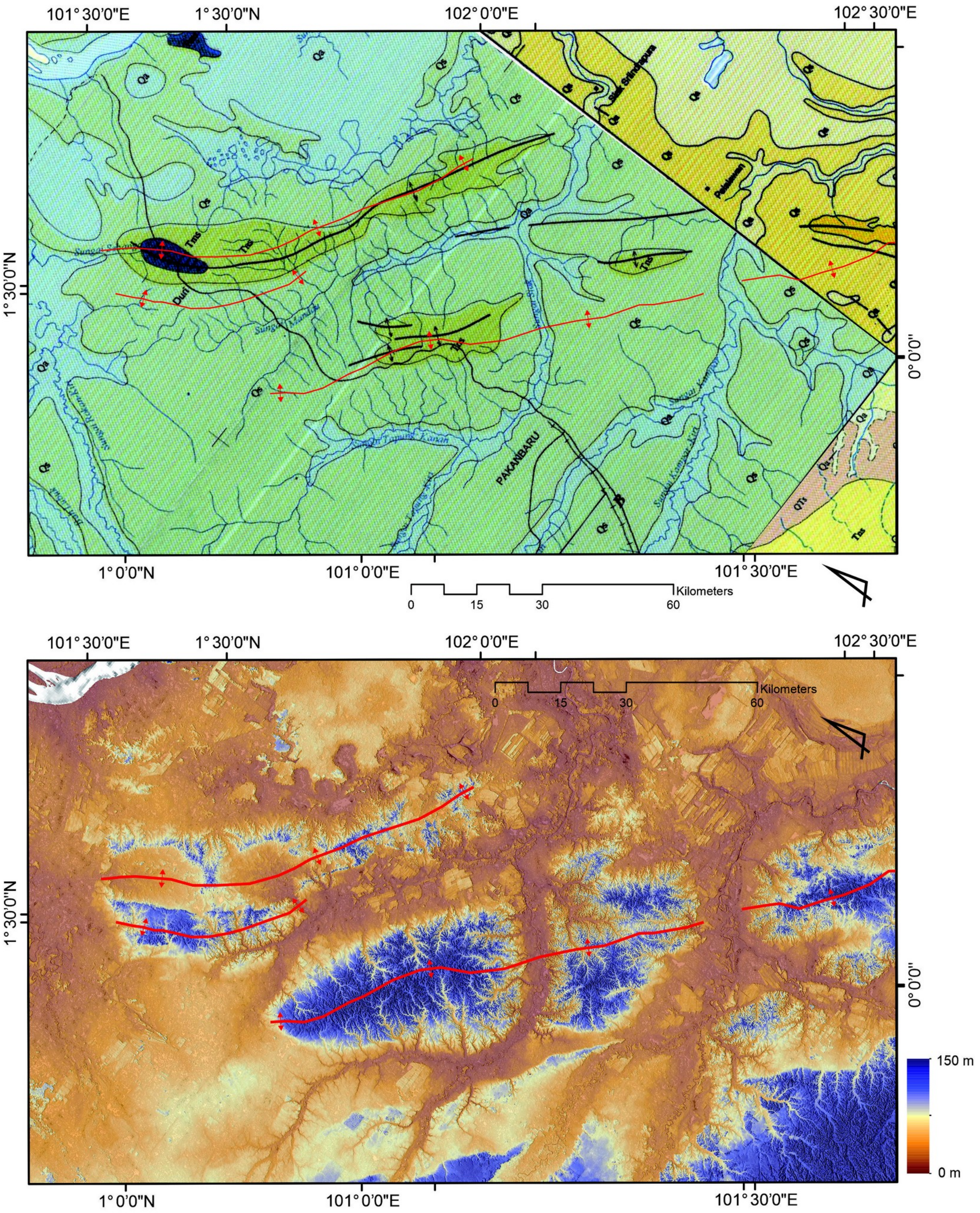

Supplementary Figure 7. *Same as Supplementary Fig. 6, but for central Sumatra. See Supplementary Figure 5 for location of map. Qa – Alluvium; Qs - Swamp Deposits; QTs - Plio-Pleistocene Non Marine Sediments; Tns - Mio-Pliocene Transitional Sediments; Tms (dark green) - Miocene Shallow Marine Sediments. Basemaps from Gafoer et al. (1996) [29].*

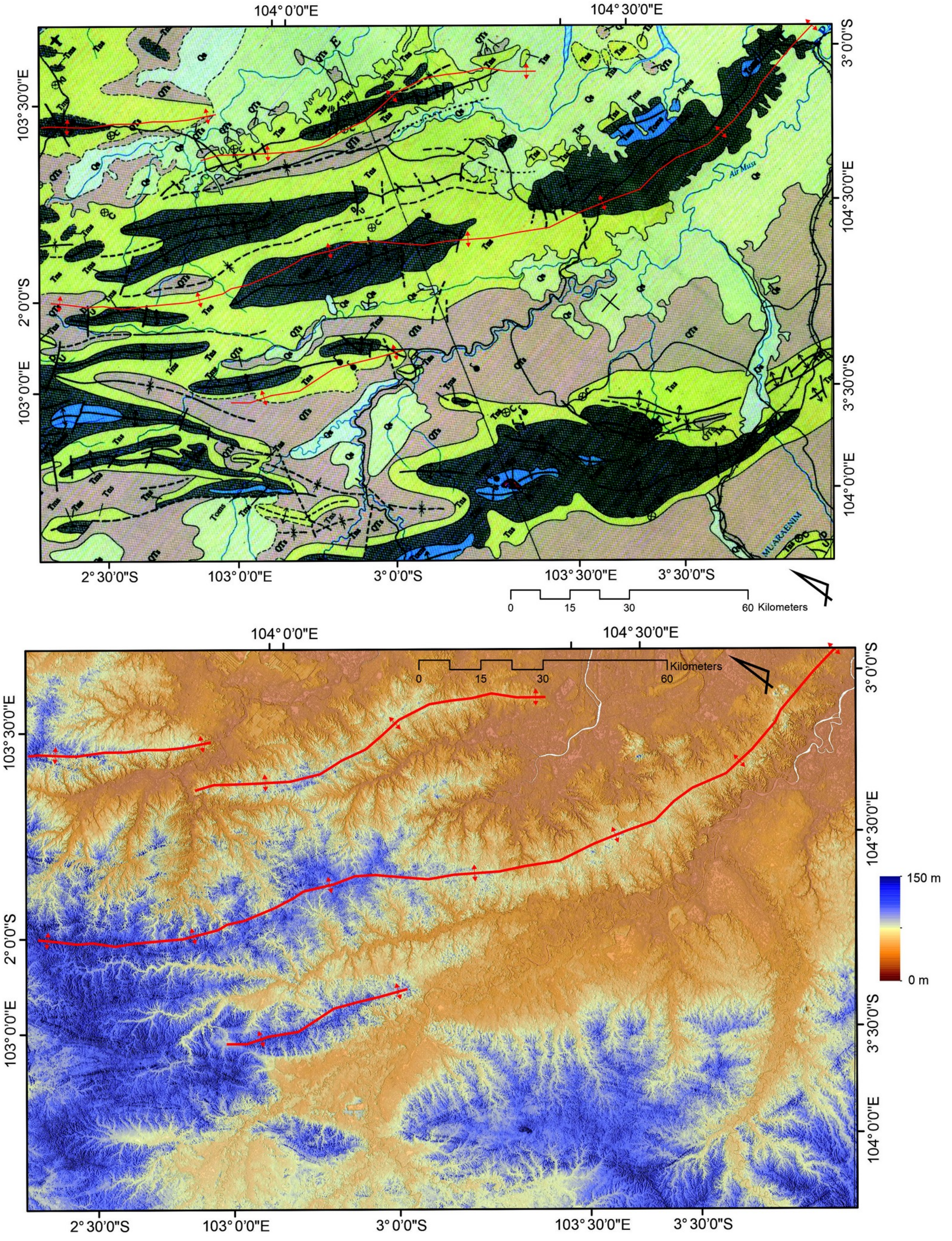

Supplementary Figure 8. *Same as Supplementary Figs. 6 and 7, but for southern Sumatra (geological map from Gafoer et al. 1992 [28]). See Supplementary Figure 5 for location of map. Qa – Alluvium;* Qs - Swamp *Deposits; QTs - Plio-Pleistocene Terrestrial Sediments; Tns - Mio-Pliocene Transitional Sediments; Tms (dark green)- Miocene Shallow Marine Sediments; Toms - Oligo-Miocene Transgressive Sediment. Basemap from Mangga et al. (1996) [51].*

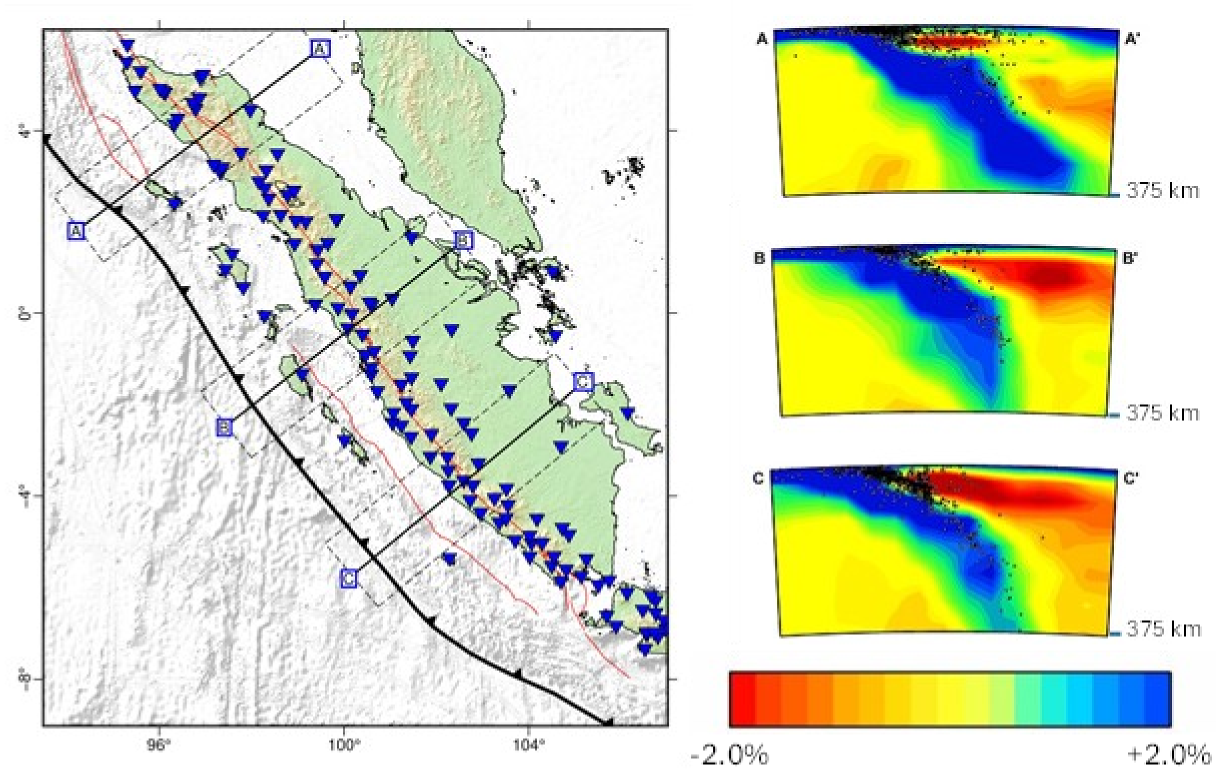

Supplementary Figure 9. *Distribution of BMKG stations (left), and associated seismic tomographic images for vertical cross sections A-A', B-B' and C-C' (right) discussed in the text. Vp perturbations relative to ak135 [37] are taken from the tomographic model by Pesicek et al. (2010) [52]. Open dots depict earthquake hypocenters of M $\geq$ 5.5 based on the ISC_EHB catalog, projected from a distance of up to 50 km on both sides of the plane of section. The model is plotted down to a depth of 375 km. Note that the majority of BMKG stations are presently positioned along the arc, with only a few in the back-arc region. Abbreviations: Great Sumatran Fault (GSF) and back-arc thrust (BAT).*

Additional References

49. Dziewonski, A. M., Chou, T.-A. & Woodhouse, J. H. Determination of earthquake source parameters from waveform data for studies of global and regional seismicity. *J. Geophys. Res*. **86**, 2825-2852, DOI:10.1029/JB086iB04p02825 (1981).

50. Ekström, G., Nettles, M. & Dziewonski, A. M. The global CMT project 2004-2010: Centroid-moment tensors for 13,017 earthquakes. *Phys. Earth Planet. Inter*. **200**, 1-9, DOI:10.1016/j.pepi.2012.04.002 (2012).

51. Mangga, S., Samodra, H. & Soetrisno, Systematic Geological Map of Indonesia - Geological Map of Indonesia, Batam Sheet; 1:1.000.000; Geological Research and Development Centre - Ministry of Energy and Mineral Resources (ESDM) (1996)

52. Pesicek, J. D., Thurber, C. H., Widiyantoro, S., Zhang, H., DeShon, H. R. & Engdahl, E. R. Sharpening the tomographic image of the subducting slab below Sumatra, the Andaman Islands and Burma, *Geophys. J. Intl.* **182**, 433–453, https://doi.org/10.1111/j.1365-246X.2010.04630.x (2010).
